# Effects of time-restricted feeding in weight loss, metabolic syndrome and cardiovascular risk in obese women

**DOI:** 10.1186/s12967-020-02687-0

**Published:** 2021-01-06

**Authors:** Jéssica D. Schroder, Hugo Falqueto, Aline Mânica, Daniela Zanini, Tácio de Oliveira, Clodoaldo A. de Sá, Andréia Machado Cardoso, Leandro Henrique Manfredi

**Affiliations:** 1 Medical School, Federal University of Fronteira Sul, UFFS, SC 484 - Km 02, Fronteira Sul, Chapecó, Santa Catarina 89815-899 Brazil; 2 Graduate Program in Biomedical Sciences, Federal University of Fronteira Sul, UFFS, SC 484 - Km 02, Fronteira Sul, Chapecó, Santa Catarina Brazil; 3 Health Science Department, Community University of the Region of Chapecó (UNOCHAPECÓ), Chapecó, SC Brazil

**Keywords:** Time-restricted feeding, Intermittent fasting, Weight loss, Metabolic syndrome, Anthropometry, Obesity, Cardiovascular risk

## Abstract

**Background:**

The increasing prevalence of overweight and obesity among the worldwide population has been associated with a range of adverse health consequences such as Type 2 diabetes and cardiovascular diseases. The metabolic syndrome (MetS) is a cluster of cardiometabolic abnormalities that occur more commonly in overweight individuals. Time-restricted feeding (TRF) is a dietary approach used for weight loss and overall health. TRF may be an option for those subjects who struggle with extreme restriction diets with foods that generally do not belong to an individual's habits.

**Objective:**

The purpose of this study was to determine the effect of TRF on body composition and the association of weight loss with metabolic and cardiovascular risks in obese middle-aged women.

**Methods:**

A non-randomized controlled clinical trial was performed over 3 months in obese women (TRF group, n = 20, BMI 32.53 ± 1.13 vs. Control n = 12, BMI 34.55 ± 1.20). The TRF protocol adopted was 16 h without any energy intake followed by 8 h of normal food intake.

**Main outcomes and measures:**

Anthropometric measurements, body composition, blood biomarkers, cardiovascular risk in 30 years (CVDRisk30y), and quality of life were evaluated at baseline and after the 3 months.

**Results:**

TRF was effective in reducing weight (~ 4 kg), BMI, % of body fat (%BF), waist circumference from baseline without changes in blood biomarkers associated with MetS. TRF promoted a reduction in CVDRisk30y (12%) wich was moderately correlated with %BF (r = 0.62, n = 64, p < 0.001) and %MM (r = − 0.74, n = 64, p < 0.001).

**Conclusions:**

TRF protocol reduces body weight without changes in biomarkers related to MetS. In addition, the anthropometric evaluation that predicts %BF and %MM could be used as an approach to follow individuals engaged in the TRF regimen since they correlate with cardiovascular risk.

## Background

Obesity is the major risk factor in the development of type 2 diabetes mellitus (T2DM), with approximately 90% of the population with T2DM being overweight or obese [1]. Another concern of obesity is the increased risk of cardiovascular disease (CVD), such as high blood pressure, atherosclerosis, acute myocardial infarction, heart failure [2]. Abdominal circumference above 102 cm in the case of men and above 88 cm in the case of women qualifies as central obesity and involves increased cardiovascular risk [3]. Both T2DM and CVD are the outcomes of a complex organic dysfunction known as metabolic syndrome (MetS) [4].

Weight loss by only 5–10% is known to improve metabolic outcomes such as glycaemic, lipid profile, and blood pressure [5]. In addition, weight loss interventions promote the reduction in all-cause premature mortality in adults with obesity [6]. Time-restricted feeding (TRF), defined by periods of severe or complete energy restriction followed by periods of habitual eating, has been suggested as a potential strategy of weight loss because it offers a reduced burden of dietary restriction and may, therefore, be more acceptable by some individuals [7]. In fact, adherence to daily energy restriction decreases after 1 month and continues to decline thereafter [8].

Although TRF is a well-known strategy to lose weight [9, 10], improvement in insulin sensitivity, blood pressure, and oxidative stress are seen even without weight loss in human studies [11]. Several randomized controlled trials (RCTs) have reported that weight loss reduces mortality in obese individuals [6]. Therefore, there is an inconsistency of whether or not weight loss is associated with changes in classical blood biomarkers such as glycemia and lipid profile. Since obesity has multifactorial factors, it is feasible to consider that blood biomarkers should be linked with body composition evaluation to follow the process of weight loss.

Despite growing bodies of studies, TRF is still a matter of debate among clinicians due to different approaches and the uncertainties of its impact on whole-body function, i.e., kidney, liver, lipid profile, hypophysis-thyroid axis, among others. Herein, weight loss was associated with blood markers of metabolic syndrome and cardiovascular risk.

It is known that the extended morning fasting period observed in TRF does not cause compensatory intake during an ad libitum lunch nor does it increase appetite during the afternoon [12]. Therefore TRF could be an alternative to those individuals who struggle with restrictive diets which changes substantially their daily habits. The purpose of this study was to determine the effect of TRF on body composition and the association of weight loss with metabolic and cardiovascular risks in obese middle-aged women.

## Methods

### Subjects

**Eligibity criteria for participation**

Obese women were included (BMI ≥ 30 kg/m^2^) in this study. Physically inactive women were excluded (less than 150 min of moderate or less than 75 min of intense physical activity *per week*). Women with non-communicable diseases other than T2DM and hypertension were excluded and individuals who were using medications other than birth control pill were also excluded.

A non-randomized controlled clinical trial on TRF was performed over 3 months in obese women. Volunteers were recruited by social networks of people inserted in the community and instant messaging applications. Outcomes were assessed at baseline and after 3 months. The control group was recruited by social media as well but participants were informed that they would be engaged in diet habit research. Participants in the TRF group were asked to continue their regular nutritional habits during the non-fasting hours while the control group was instructed to maintain their habitual nutrition throughout the whole period. The protocol was approved by the Committee of Ethical Research from the Federal University of Fronteira Sul (protocol number 2.819.932), and written informed consent was obtained from all participants. Trial registration: *ensaiosclinicos.gov.br* (10051). Registered 16 June 2020—retrospectively registered.

### Time-restricted feeding

TRF is based on the manipulation of timing (fasting) that aims the energy intake abstention [9]. The TRF protocol adopted here was a fasting period (no energy intake whatsoever) of 16 h (8 pm to 12 pm) and ad libitum feeding for 8 h (12 pm to 8 pm). The protocol was performed 7 days per week for 3 months. All participants received daily a reminder through instant messaging that informed the time to stop eating and the time in which food was allowed. In addition, periodic meetings were promoted (one every 15 days) for participants to share their experience and receive support from physicians. The control group was instructed to maintain the same dietary and living habits.

**Main outcomes and measurements**

The primary outcome was the effect of intermittent fasting on body weight and composition after 3 months compared to baseline values. Secondary outcomes were the characterization of metabolic risk factors and their association with weight loss. We also measured the cardiovascular risk of the participants at baseline and post 3 months.

### Anthropometric measures and body composition

Weight was collected using the digital weight balance (Urano, PS 180). Height was measured by the wall stadiometer and waist circumference (upper edge of the iliac crest) with fine metric (Sanny, fiberglass tape). Body mass index (BMI) values were calculated from these measurements. For body composition analysis, we used anthropometric prediction equations, which were validated by the National Health and Nutrition Examination Survey (NHANES). We used the equation of Lee et al. [13] [SE (R^2^) = 2.44 (0.93)] and Heymsfied et al. [14] [SE (R^2^) = 1.6 (0.87)] with weight, height, waist circumference (WC) and race/ethnicity data to calculate total body fat mass (FM) and total skeletal muscle mass (MM), respectively. The values of total FM were divided by the total body weight and height^2^, to obtain the body fat percentage (%BF) and fat mass index (FMI), respectively. The values of MM were divided by total body weight, BMI and height^2^, to obtain the body skeletal muscle percentage (%MM), muscle mass related to BMI (MM/BMI), and muscle mass index related to height (SMI_height_), respectively. All anthropometric measurements were performed thrice by one subject. If an error greater than 1% among the measurements was detected, a new measurement was taken.

### Blood pressure measurement

Systolic blood pressure (SBP) and diastolic blood pressure (DBP) were measured using a mercury sphygmomanometer (Kenz, 600) by a single evaluator according to the European Society of Cardiology/European Society of Hypertension checklist criteria [15].

**Blood collection and analysis**

Blood samples taken from the antecubital vein at baseline and after 3 months were collected in BD Vacutainers Tubes (SST™ II Advance, REF 367953). Samples were centrifuged (4000 RPM at 4 °C using centrifuge J6-MC by Beckman), and the resultant serum was aliquoted and stored at − 80 °C.

Acid uric, albumin, bilirubin, total cholesterol, high-density lipoprotein cholesterol (HDL-c), low‐density lipoprotein cholesterol (LDL), creatinine, alkaline phosphatase, gamma-glutamyl transferase (GGT), fasting glucose, Na^+^, K^+^, reactive C protein (PCR), alanine transaminase (ALT), aspartate aminotransferase (AST), triglycerides, and urea were evaluated by AU480 Chemistry Analyzer (Beckman Coulter) following the instruction provided by the manufacturer. Estradiol, insulin, free thyroxine (T_4_), and Thyroid-stimulating hormone (TSH) were analyzed by UniCel DxI 800 Access Immunoassay System (Beckman Coulter) following the instructions and protocols provided by the manufacturer.

The homeostatic model assessment (HOMA) index was calculated by the formula: HOMA-IR = fasting plasma insulin (µU/mL) × fasting plasma glucose (mmol/L)/22.5 [16].

### Metabolic syndrome

To evaluate whether or not women presented MetS, we followed the criteria from Alberti et al. [4]. Participants were considered to have MetS when they presented three or more of the following components: WC > 80 cm (reference value for Ethnic Central and South American) [17]; triglycerides > 150 mg/dL or drug treatment for triglycerides reduction; HDL-C < 50 mg/dL or drug treatment for reduced HDL-C; SBP > 130 mmHg and/or DBP > 85 mmHg or antihypertensive drug treatment and fasting glucose > 100 mg/dL or drug treatment for elevated glucose. Participants were classified in 0–5 score according to the MetS criteria.

### 30-year cardiovascular disease risk evaluation

We have used the Framingham Heart Study, which provided an estimation of the 30-year CVD risk (CVDRisk30y) for each individual [18]. Framingham risk scores (FRS) for CVD covers the full spectrum of CVD, including coronary heart disease, peripheral vascular disease, stroke, and heart failure [19]. The online FRS calculator is user-friendly and free of cost (https://framinghamheartstudy.org/fhs-risk-functions/cardiovascular-disease-30-year-risk). The online calculation requires information on the age, gender, systolic blood pressure (at the time of the interview), treatment for hypertension (yes/no), diabetes (yes/no), smoking (yes/no), and BMI (kg/m^2^).

### Quality of Life and Mini-Mental State Examination

The Quality of Life (QOL) assessed by WHOQOL-bref questionnaire has been translated and validated in Brazil [20]. The abbreviated WHOQOL-bref provides scores for four domains related to QOL: physical health, psychological, social relationships, and environment. Also, a self-perception of quality of life is measured by this questionnaire [21]. The WHOQOL-bref consists of 26 items rated on a 5-point Likert scale. The response options range from 1 (very dissatisfied/very poor) to 5 (very satisfied/very good) [20]. The scores are transformed and vary from 0 to 100, with higher scores representing better QOL [22]. The Mini-Mental State Examination (MMSE) evaluates the cognition health and it has been translated and validated in Brazil [23]. The test provides a score of 0–30. Given the low levels of education among older adults in Brazil, specific cut-off points are used based on the schooling level of the older adults: 13 for illiterate people, 18 for those with 1–11 years of schooling, and 26 for those with more than 11 years of schooling [23]. This questionnaire was used as a tool to characterize the participants and assess whether or not they would fully understand the protocol in order to follow it.

### Statistical analysis

This study was conducted as an exploratory pilot study with the recruitment of women who were engaged in performing the protocol. Therefore, a sample size calculation did not seem possible. Results are presented as mean ± standard error of mean (SEM). Shapiro–Wilk’s W test was conducted to assess the normality. In order to reduce the influence of within-group variability, a univariate test of significance (ANCOVA) was performed. Levene’s test of homogeneity for equal variances was performed. For multiple comparisons (sensitivity analyses) Bonferroni correction of p-values was used. We fixed as dependent variable the post value of the clinical exams/anthropometric measurements for each group and the baseline values of the outcomes were adopted as a covariate. TRF group vs. Control group were assumed as categorical predictors. A paired Student t-test was performed between baseline and 3 months for each group. A Chi‐square test was used to compare the difference in MetS between groups. A post hoc power was performed using effect size Cohen’s d = 0.5. We calculated that this study had a power of 75%. The correlation CVDRisk30y and %BF, and CVDRisk30y and %MM were performed by Pearson´s correlation. P < 0.05 was adopted as significant differences. Statistical analyses were made with the statistical software package Statistical Package for the Social Science (SPSS), version 26.0.

## Results

**Fig. 1:**
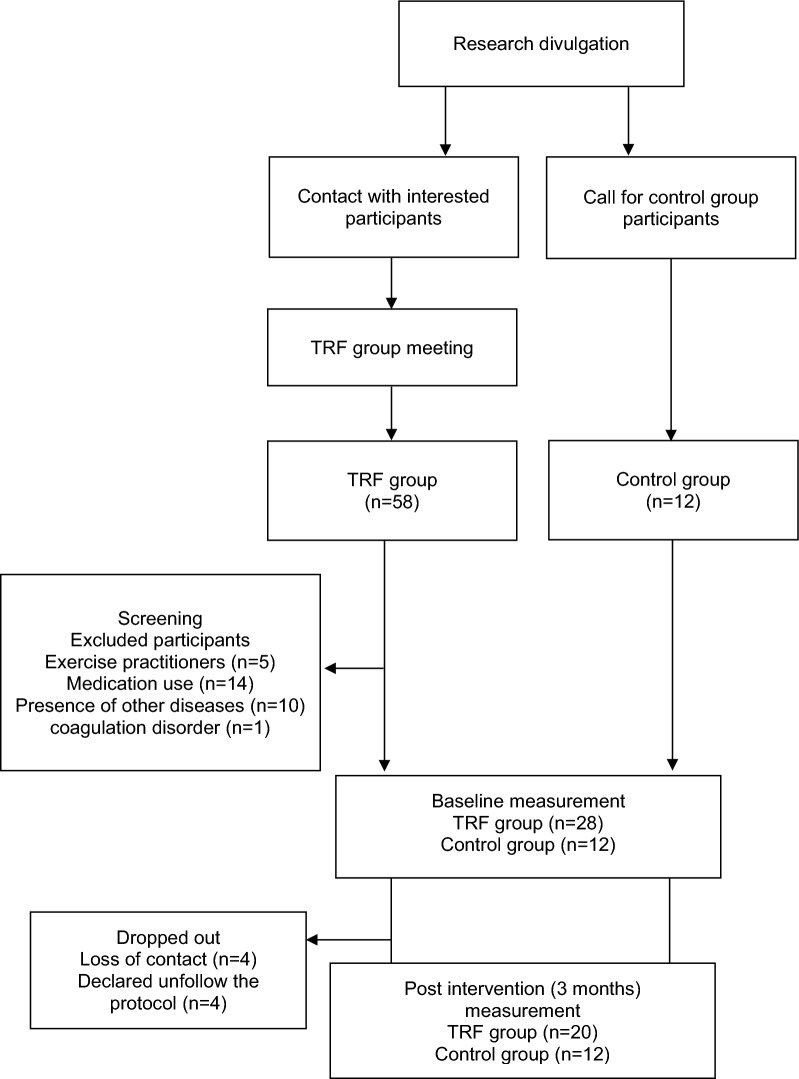
Flow chart from the study design

Fifty-eight interested subjects contacted our staff willing to engage in the intermittent fasting protocol, twenty-eight subjects after screening met the eligibility criteria, and one subject had to be excluded due to a coagulation disorder (Fig. 1). A total of twenty obese women (n = 20) voluntarily adopted the fasting protocol, while twelve obese women (n = 12) were enrolled in the control group. All women who were enrolled in this study attended the following criteria: sedentarily, non-communicable diseases, and physically inactive. Figure 1 shows the flowchart of the study with the dates that each event occurred.

### Sample characteristic

Participant’s baseline characteristics are provided in Table 1. No significant between-group differences were observed at baseline in any measurement performed. Baseline values for participants of both groups reflect middle-aged women with obesity with no cognition impairment.

**Table 1:** Subject characteristics at baseline

	TRF group (n = 20)	Control Group (n = 12)	P-value
	Baseline	Baseline	
Age	36.6 ± 1.6	42.3 ± 3.5	0.10
Height (cm)	159.8 ± 1.6	158.8 ± 1.3	0.66
Weight (kg)	83.62 ± 3.95	87.14 ± 3.25	0.54
BMI (kg/m^2^)	32.53 ± 1.13	34.55 ± 1.20	0.25
Fat mass (kg)	36.91 ± 2.30	39.74 ± 2.00	0.41
WC (cm)	101.13 ± 2.42	105.68 ± 3.11	0.26
%BF	43.61 ± 0.69	45.38 ± 0.69	0.10
FMI (kg/m^2^)	14.33 ± 0.72	15.71 ± 0.76	0.22
MM (kg)	21.59 ± 0.93	21.59 ± 0.70	0.99
%MM	26 ± 0.00	25 ± 0.00	0.01
SMI_height_ (kg/m^2^)	8.40 ± 0.25	8.51 ± 0.23	0.75
MM/BMI (kg/kg/m^2^)	0.66 ± 0.02	0.63 ± 0.02	0.15
Insulin (mU/L)	9.2 ± 0.9	10.3 ± 1.8	0.54
Fasting glucose (mg/dL)	86.2 ± 1.2	91.6 ± 3.0	0.07
HOMA-IR	1.88 ± 1.91	2.35 ± 0.44	0.19
SBP (mmHg)	126.7 ± 3.4	134.5 ± 7.3	0.28
DBP (mmHg)	86.9 ± 2.1	91.3 ± 4.1	0.28
MMSE	27.0 ± 0.4	26.5 ± 1.0	0.60
CVDRisk30y (%)	32.1 ± 7.3	15.6 ± 1.8	*< 0.001*

Results are presented as mean ± SEM

*%BF* body fat percentage, *%MM* body skeletal muscle percentage, *BMI* body mass index, *CVDRisk30y* 30 year CVD risk,* DBP* diastolic blood pressure, *FMI* fat mass index, *MAP* median arterial pressure *MetS* metabolic syndrome, *MM* muscle mass, *MM/BMI* muscle mass related to BMI, *MMSE* mini-mental state examination, *SBP* systolic blood pressure, *SMI* skeletal muscle index, *WC* waist circumference

### Outcome measurements

Table 2 summarizes the body weight and composition outcomes. The TRF group exhibited a decrease in body weight, BMI, FM, WC, %BF, FMI, MM, SMI_height_, SBP, and DBP. Despite changes in body weight and body composition, there were no significant changes in blood biomarkers associated with metabolic and cardiovascular risk.

**Table 2:** Results of anthropometric analysis and blood biomarkers in the time-restricted-feeding and control group at baseline and after 3 months

	TRF group (n = 20)			Control group (n = 12)				∆ P-value
	Baseline	3 months	Δ TRF *t *test	Baseline	3 months	Δ Control *t *test	∆ (TRF-control)	
Weight (kg)	83.62 ± 3.95	80.24 ± 3.87	*< 0.001*	87.14 ± 3.25	88.49 ± 3.04	0.007	− 4.856	*< 0.001*
BMI (kg/m^2^)	32.53 ± 1.13	31.19 ± 1.11	*< 0.001*	34.55 ± 1.20	35.00 ± 1.10	0.078	− 1.886	*< 0.001*
Fat mass (kg)	36.91 ± 2.30	34.74 ± 2.24	*< 0.001*	39.74 ± 2.00	40.59 ± 1.95	0.050	− 3.125	*< 0.001*
WC (cm)	101.13 ± 2.42	97.15 ± 2.21	*0.001*	105.68 ± 3.11	106.95 ± 2.81	0.412	− 6.041	*< 0.001*
%BF	43.61 ± 0.69	42.71 ± 0.73	*< 0.001*	45.38 ± 0.69	45.73 ± 0.67	0.079	− 1.218	*< 0.001*
FMI (kg/m^2^)	14.33 ± 0.72	13.47 ± 0.71	*< 0.001*	15.71 ± 0.76	16.10 ± 0.76	0.060	− 1.301	*< 0.001*
MM (kg)	21.59 ± 0.93	20.91 ± 0.92	*< 0.001*	21.59 ± 0.70	21.78 ± 0.68	0.121	− 0.901	*< 0.001*
%MM	25.9 ± 1.1	26.2 ± 1.2	0.11	24.8 ± 1.3	24.7 ± 1.3	0.349	0.397	0.11
SMI (kg/m^2^)	8.40 ± 0.25	8.13 ± 0.24	*< 0.001*	8.51 ± 0.23	8.63 ± 0.23	0.128	− 0.394	*< 0.001*
MM/BMI (kg/kg/m^2^)	0.66 ± 0.02	0.67 ± 0.02	0.09	0.63 ± 0.02	0.62 ± 0.02	0.220	0.011	0.05
Insulin (mU/L)	9.2 ± 0.9	9.1 ± 1.0	0.86	10.3 ± 1.8	13.8 ± 3.2	0.133	− 3.494	0.07
Glucose (mg/dL)	86.2 ± 1.2	88.0 ± 1.4	0.25	91.6 ± 3.0	93.6 ± 4.5	0.458	− 0.336	0.91
HOMA-IR	1.88 ± 1.91	1.90 ± 0.22	0.88	2.35 ± 0.44	3.27 ± 0.86	0.117	− 0.717	0.13
SBP (mmHg)	126.7 ± 3.4	121.3 ± 3.1	*0.03*	134.5 ± 7.3	128.0 ± 4.0	0.227	− 2.542	0.48
DBP (mmHg)	86.9 ± 2.1	83.5 ± 2.1	0.06	91.3 ± 4.1	86.5 ± 4.1	0.167	0.111	0.97
Na^+^ (mEq/L)	141.5 ± 0.7	144.0 ± 0.8	*0.02*	142.7 ± 0.6	140.5 ± 1.8	0.336	3.268	0.10
K^+^ (mEq/L)	4.3 ± 0.08	4.3 ± 0.10	1.000	4.3 ± 0.07	4.3 ± 0.17	0.769	0.045	0.80
Frutosamine (µmol/L)	240.1 ± 5.6	242.5 ± 3.4	0.52	236.5 ± 5.2	240.3 ± 6.2	0.260	0.128	0.10
TC (mg/dL)	205.9 ± 8.3	214.7 ± 9.7	0.18	195.1 ± 11.3	188.5 ± 13.3	0.307	15.818	0.11
HDL-C (mg/dL)	60.5 ± 2.1	61.1 ± 2.3	0.73	56.6 ± 5.1	53.7 ± 4.8	0.201	4.177	0.14
LDL-C (mg/dL)	120.0 ± 6.5	127.4 ± 7.8	0.20	112.8 ± 7.3	111.1 ± 9.1	0.715	9.468	0.26
Cr (mg/dL)	0.70 ± 0.03	0.69 ± 0.02	0.79	0.67 ± 0.18	0.69 ± 0.03	0.475	− 0.007	0.82
Triglycerides (mg/dL)	144.0 ± 18.0	131.9 ± 17.6	0.18	148.1 ± 28.9	130.9 ± 24.8	0.228	4.307	0.77
CRP (mg/L)	6.3 ± 1.5	6.6 ± 1.4	0.86	9.1 ± 2.8	9.4 ± 2.2	0.889	− 1.599	0.47
Urea (mg/dL)	27.8 ± 1.4	27.6 ± 2.1	0.90	32.8 ± 1.4	32.3 ± 2.4	0.801	− 1.259	0.70
Total Bilirubin (mg/dL)	0.46 ± 0.07	0.42 ± 0.06	0.35	0.35 ± 0.03	0.35 ± 0.02	0.902	− 0.008	0.88
Indirect bilirubin (mg/dL)	0.37 ± 0.06	0.34 ± 0.05	0.38	0.28 ± 0.03	0.28 ± 0.02	0.922	− 0.002	0.97
Direct bilirubin (mg/dL)	0.09 ± 0.01	0.08 ± 0.01	0.30	0.07 ± 0.007	0.07 ± 0.005	0.461	− 0.005	0.65
ALP (U/L)	69.8 ± 4.9	65.5 ± 3.9	0.26	81.6 ± 9.5	69.9 ± 8.1	0.340	− 0.946	0.90
GOT (U/L)	19.7 ± 1.1	17.7 ± 1.6	0.14	18.2 ± 1.5	18.0 ± 1.3	0.916	− 1.307	0.53
ALT (U/L)	18.6 ± 2.0	15.0 ± 1.9	*0.002*	16.8 ± 2.1	17.1 ± 1.7	0.919	− 3.219	0.12
GGT (U/L)	23.0 ± 2.9	19.4 ± 2.1	0.06	29.3 ± 8.7	27.8 ± 5.4	0.751	− 4.818	0.05
Uric Acid (mg/dL)	4.7 ± 0.2	4.7 ± 0.2	0.93	5.0 ± 0.4	5.3 ± 0.4	0.358	− 0.398	0.27
TSH (µUI/mL)	3.06 ± 0.89	2.51 ± 0.39	0.09	2.65 ± 0.46	2.04 ± 0.21	0.130	0.126	0.71
T_4_ (ng/dL)	0.82 ± 0.03	0.86 ± 0.02	0.15	0.81 ± 0.02	0.83 ± 0.04	0.426	0.027	0.41
Total Protein (g/dL)	7.3 ± 0.1	7.3 ± 0.1	0.84	7.3 ± 0.1	7.0 ± 0.2	0.251	0.335	0.12
Albumin (g/dL)	4.2 ± 0.1	4.3 ± 0.1	0.70	4.2 ± 0.1	4.0 ± 0.2	0.195	0.307	0.18
Globulin (g/dL)	3.1 ± 0.1	3.0 ± 0.1	0.77	3.1 ± 0.1	3.0 ± 0.1	0.336	0.045	0.79
Estradiol (pg/mL)	128.2 ± 18.1	98.6 ± 15.7	0.17	67.9 ± 18.4	59.4 ± 10.6	0.565	24.190	0.31

Results are presented as mean ± SEM. A paired t-test was performed between baseline and post-intervention in each group. ANCOVA was performed to detect changes in effect between groups

*ns* not significant,* %BF* body fat percentage, *%MM* body skeletal muscle percentage, *ALP* alkaline phosphatase, *ALT* alanine aminotransferase, *AST* aspartate aminotransferase, *BMI* body mass index, *CRP* C-reactive protein, *DBP* diastolic blood pressure, *FMI* fat mass index, *GGT* gamma-glutamyl transferase, *HDL-C* high-density lipoprotein-cholesterol; *K*^+^ blood potassium, *LDL-C* low-density lipoprotein-cholesterol, *MM* muscle mass, *MM/BMI* muscle mass related to BMI, *Na*^+^ plasmatic sodium, *SBP* systolic blood pressure, *SMI* skeletal muscle index, *TC* total cholesterol, *TSH* thyroid-stimulating hormone, *T*_*4*_ free thyroxin, *WC* waist circumference

**Fig. 2:**
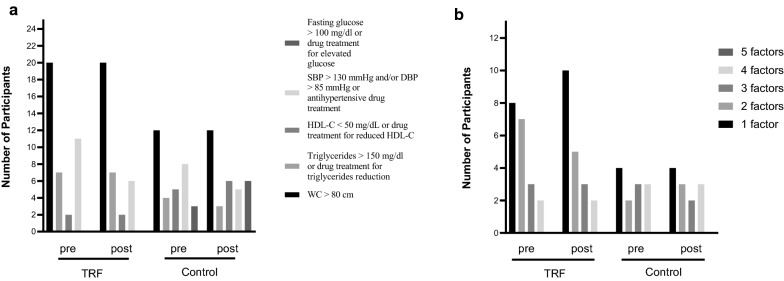
**a** Metabolic Syndrome risk factors and the number of individuals that have each factor in TRF and control groups in pre and post-intervention. **b** The number of participants who have 1, 2, 3, 4, or 5 risk factors

Figure 2a shows the criteria and the number of participants with the respective risk of MetS at baseline and post-intervention. According to Alberti et al. [4] criteria, the number of individuals with metabolic syndrome remained the same in both groups after the period of the study. Figure 2b shows the number of women who presented 1, 2, 3, 4, or 5 risk factors at baseline (pre) and post-intervention.

### CVDRisk30y and its correlation to body fat and muscle mass

**Fig. 3:**
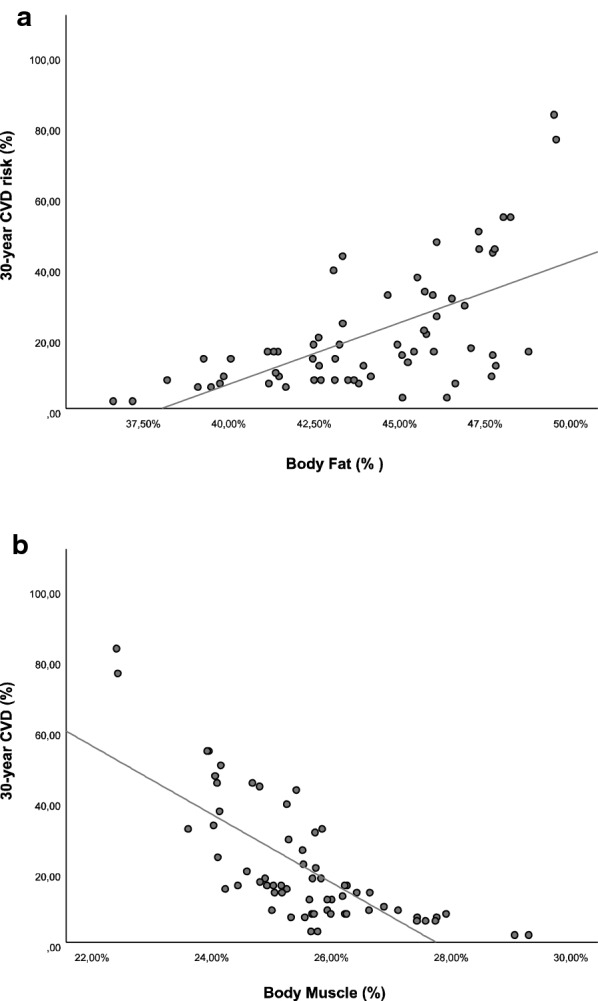
Correlation between percentual of body fat (%BF) and the risk of cardiovascular disease in 30 years (CVRisk30y) and the percentage of body skeletal muscle mass (%MM) and the risk of cardiovascular disease in 30 years (CVRisk30y)

We have conducted an analysis of CVDRisk30y based on The Framingham Heart Study. In the TRF group, there was a ~ 12% reduction (%, 15.6 ± 1.8 pre vs. 13.8 ± 1.8 post-intervention, P < 0.05) in the probability of general cardiovascular events to happen. There was no change in CVDRisk30y in the control group (%, 32.1 ± 7.2 pre vs. 31.1 ± 6.7 post-intervention). To evaluate anthropometric changes with cardiovascular risk other than the ones predict in the Framingham Heart Study, we correlated all anthropometric indexes to CVDRisk30y (Additional file 1: Table S1). The best predictors of CVDRisk30y were %BF (r = 0.62, n = 64, p < 0.001) and %MM (r = − 0.74, n = 64, p < 0.001), and they are shown in Fig. 3.

### Quality of Life Questionnaire

**Fig. 4:**
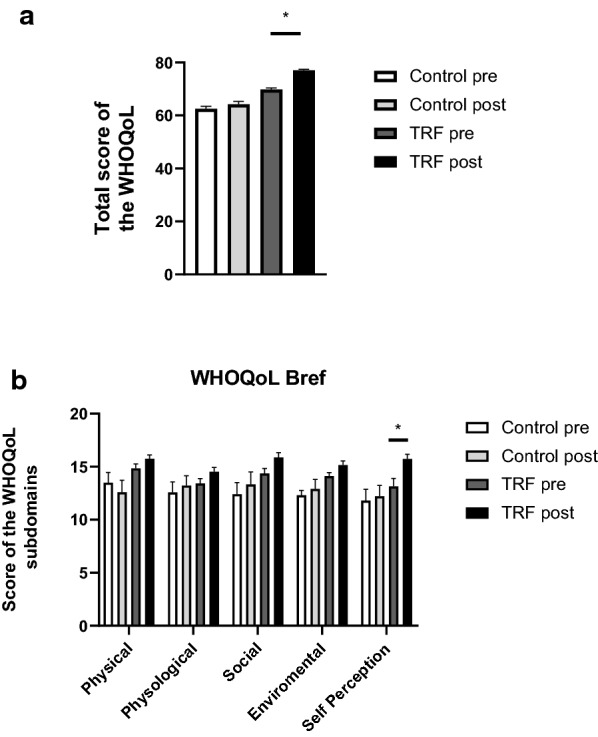
Total (**a**) and subdomains (**b**) scores of the WHOQoL. Values represent mean ± SEM and are represented as a percentage. (pre refers before intervention and post after intermittent fasting protocol). *P < 0.05 vs. IF pre

The total score for WHOQOL-bref questionnaire and the scores by subdomain are represented in Fig. 4a, b, respectively. Participants after TRF protocol obtained a higher score when compared to baseline values, and this fact was due to a self-perception of a better quality of life, as seen in Fig. 4b.

## Discussion

In this study, the short-term effects of TRF on metabolic, hormonal, and anthropometric parameters were evaluated. TRF has shown to be an effective protocol to promote weight loss, anthropometric, and body composition changes, but did not show significant changes in blood biomarkers associated with metabolic and cardiovascular risk. Our findings differ from previous results in which TRF promotes changes in blood exams and metabolic parameters (glycemia, HDL, LDL, cholesterol, among others) [11, 24]. The TREAT Randomized Clinical Trial reported similar findings with no changes in fasting insulin and glycemia in a 16:8-h time-restricted eating protocol in overweighted adults [25].

It is widely accepted that obesity is associated with all-cause mortality and the development of cardiovascular events in mid-age adults [26, 27]. Also, overweight per se, without the presence of MetS, is an independent factor related to increased mortality and cardiovascular events [28–30]. In our study, 75% of obese women in the TRF group did not exhibit MetS at baseline measurements (presence of 3 or more risk factors, according to Fig. 2b). Therefore they were considered to have obesity since their BMI was greater than 30 kg/m^2^ without any change in metabolic biomarkers. This aspect is extremely relevant once any significant change in blood exams (glucose, triglycerides, HDL-c, etc.) may not appear before three months, confirming that obesity first-line treatment should aim weight-loss strategies. Evidence in humans suggests that the benefits of TRF are due mostly or only to weight loss [8, 31–33]. The variation among participants with distinct risk factors may be taken into account for different responses to the TRF protocol. Further studies are necessary to evaluate whether or not these factors impact on individual responses to TRF.

Except for weight and height, WC was the only direct anthropometric measurement performed in this study and it was reduced with TRF. It is well-known that WC is an indicator of visceral adiposity and a predictor of morbidity and mortality [34]. RCTs reveal that reduction in WC promoted by lifestyle change is associated with a significant decrease in cardiometabolic risk independently from gender or age [35, 36]. In our study, a significant positive correlation was seen between WC and cardiovascular risk, WC and %BF, and a significant negative correlation between WC and %MM. Therefore, WC measurement and %BF and%MM calculation by the prediction methods used here are valuable approaches for monitoring the results of a TRF protocol.

Our study has shown that TRF is a benefic dietary intervention that leads to an increase in self-reported quality of life, which can be explained by weight loss [37, 38]. Besides, obese women often report dissatisfaction concerning their bodies when compared to subjects with normal weight [39]. Since TRF promoted a reduction in weight and waist circumference, the improvement in the quality of life seen in these women may be indeed attributed to a self-perception of a better body image [40].

### Clinical relevance

Obesity is characterized by the accumulation of adipose tissue and an increase in body mass, which develops under a chronic positive energy balance. Therefore the reduction of this excess of adipose mass is the main goal of the clinical approach to treat obesity [41–43]. TRF can be an efficient strategy to promote weight loss in those subjects who cannot adhere to diets that alter the whole nutritional pattern of the individual’s daily food ingestion.

Most of the subjects in our study are obese but did classic biomarkers are not altered, corroborating data from populational studies [44]. Nonetheless, these individuals are targets of the deleterious effects of excess adipose tissue that will trigger MetS at any time. This study points out the importance of a more comprehensive evaluation of overweight or obese subjects, which includes anthropometric measurements.

### Limitations

This study was not a randomized controlled trial and does not include detailed nutritional aspects of the subject´s diet since we found conflicting data reported by individuals, such as an incomplete description of the amount of food ingested, the frequency of meals, and the type of foods (e.g.: what kind of rice was ingested). In addition, the dietary intake self-report can differ or underestimate the real value, offering an inconclusive and misleading analysis [45, 46]. Energy restriction is the main factor that leads to weight loss independently of the type of diet [9]. Therefore, it is feasible to assume that a reduction in total energy consumed was achieved, considering that energy expenditure has maintained constant.

A recent meta-analysis has shown a dose–response between weight loss and reduction in energy intake [47]. Therefore, one can assume that weight loss was higher in those women who had more energy balance deficit. Another concern is the protocol adherence, which can interfere in the outcomes. Since we are not able to control whether or not the subjects followed the TRF strictly for three months without any gap, explaining differences in weight loss among subjects is a hard task. Herein, 45% of the women in the TRF group did not lose more than 4% of the weight. Since it is well established that negative energy balance promotes weight loss, we have stratified our analysis to avoid misinterpretation of what event was due to protocol adherence or the individual’s response to the protocol.

## Conclusion

TRF is an effective dietary strategy to promote weight loss and to decrease WC with no remarkable changes in blood biomarkers. This can be explained by the considerable number of obese women without MetS, in which they have an excess of weight and WC, but not always altered blood biomarkers. Also, anthropometric prediction of %BF and %MM can be used as an approach to guide health professionals to evaluate and follow individuals engaged in TRF protocol since those measurements correlate with cardiovascular risk.

## Supplementary Information

**Additional file 1: Table S1.** Two-variable correlation. Pearson correlation, p-value (Sig.), and the number of cases (n) are presented in the table.

## Data Availability

We may provide all raw data to the Journal upon request. Relevant data may be provided to readers upon request.

## Abbreviations

%BF: Body fat percentage
%MM: Body skeletal muscle percentage
ALT: Alanine transaminase
BMI: Body mass index
CVD: Cardiovascular disease
CVDRisk30y: Estimation of the 30-year CVD risk
DBP: Diastolic blood pressure
FM: Fat mass
FMI: Fat mass index
FRS: Framingham risk scores
GGT: Gamma-glutamyl transferase
HDL-c: High-density lipoprotein cholesterol
HOMA: Homeostatic model assessment
LDL: Low‐density lipoprotein cholesterol
MAP: Mean arterial pressure
MetS: Metabolic syndrome
MHO: Metabolically healthy obesity
MM/BMI: Muscle mass related to BMI
MM: Muscle mass
MMSE: Mini-Mental State Examination
NHANES: National Health and Nutrition Examination Survey
PCR: Reactive C protein
QOL: Quality of Life Questionnaire
SBP: Systolic blood pressure
SE: Standard error
SEM: Standard error of mean
SMI_height_: Muscle mass index related to height
SPSS: Statistical package for the social science
T2DM: Type 2 diabetes mellitus
T_4_: Thyroxine
TRF: Time-restricted feeding
TSH: Thyroid-stimulating hormone
