# Supplementary material for: Effects of time-restricted feeding in weight loss, metabolic syndrome and cardiovascular risk in obese women
Source: J Transl Med. 2021 Jan 6;19:3. doi: 10.1186/s12967-020-02687-0 (PMC7786967; doi:10.1186/s12967-020-02687-0)
Supplement: Supplementary file 1 — Additional file 1: Table S1. Two-variable correlation. Pearson correlation, p-value (Sig.), and the number of cases (n) are presented in the table. [file 12967_2020_2687_MOESM1_ESM.ppt]

## Slide 1
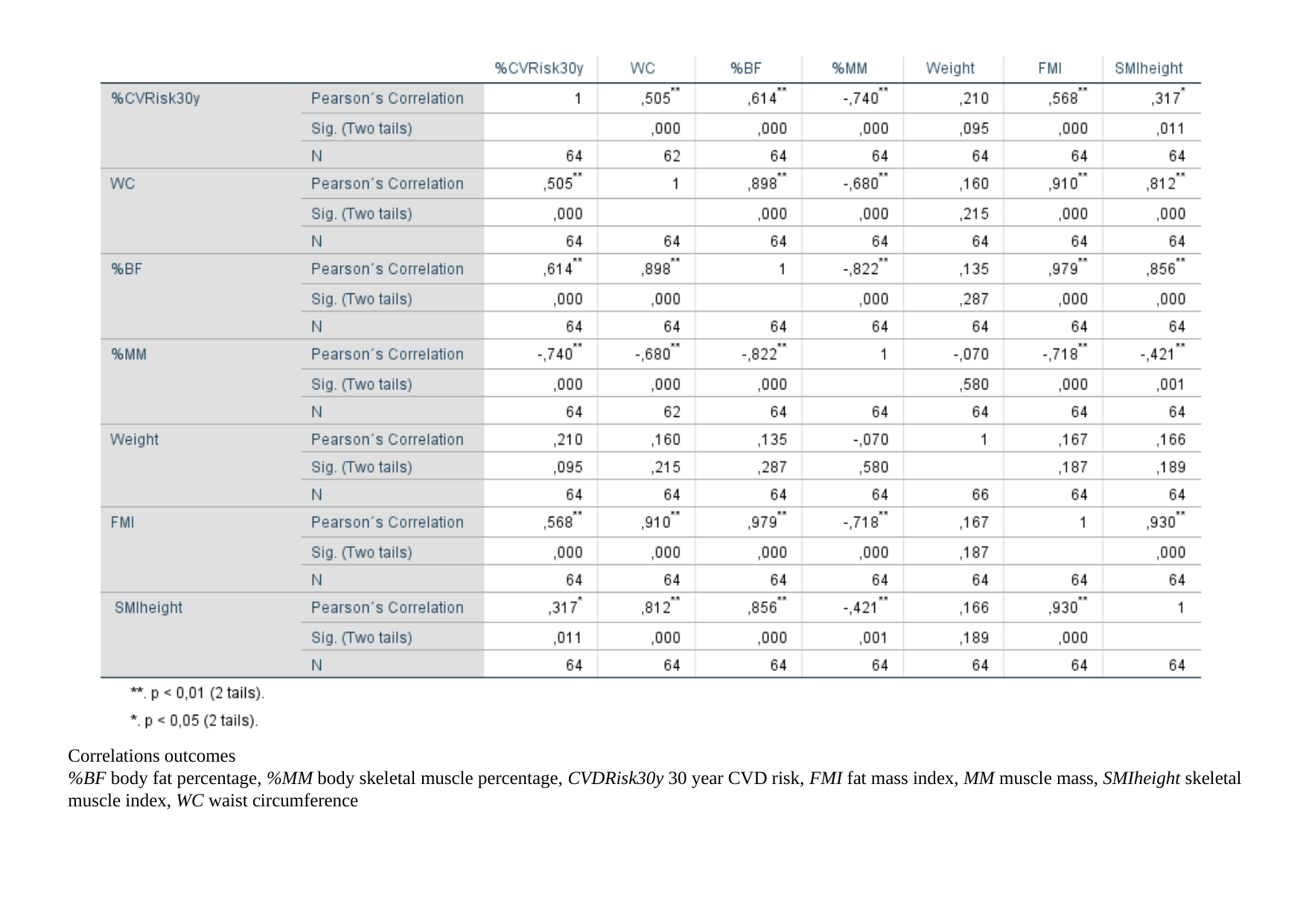

Correlations outcomes
%BF body fat percentage, %MM body skeletal muscle percentage, CVDRisk30y 30 year CVD risk, FMI fat mass index, MM muscle mass, SMIheight skeletal muscle index, WC waist circumference
